# Epimerization of Deoxynivalenol by the *Devosia* Strain A6-243 Assisted by Pyrroloquinoline Quinone

**DOI:** 10.3390/toxins14010016

**Published:** 2021-12-25

**Authors:** Hui Gao, Jiafeng Niu, Hua Yang, Zhaoxin Lu, Libang Zhou, Fanqiang Meng, Fengxia Lu, Meirong Chen

**Affiliations:** Laboratory of Enzyme Engineering, College of Food Science and Technology, Nanjing Agricultural University, Nanjing 210095, China; gh2018@njau.edu.cn (H.G.); njf2021@stu.njau.edu.cn (J.N.); yanghua@njau.edu.cn (H.Y.); fmb@njau.edu.cn (Z.L.); zhoulb@njau.edu.cn (L.Z.); mfq@njau.edu.cn (F.M.)

**Keywords:** deoxynivalenol, epimerization, pyrroloquinoline quinone, *Devosia*, *Pseudomonas*

## Abstract

Deoxynivalenol (DON) is a secondary metabolite produced by several *Fusarium* species that is hazardous to humans and animals after entering food chains. In this study, by adding cofactors, the *Devosia* strain A6-243 is identified as the DON-transforming bacteria from a bacterial consortium with the ability to biotransform DON of *Pseudomonas* sp. B6-24 and *Devosia* strain A6-243, and its effect on the biotransformation process of DON is studied. The *Devosia* strain A6-243 completely biotransformed 100 μg/mL of DON with the assistance of the exogenous addition of PQQ (pyrroloquinoline quinone) within 48 h and produced non-toxic 3-epi-DON (3-epi-deoxynivalenol), while *Pseudomonas* sp. B6-24 was not able to biotransform DON, but it had the ability to generate PQQ. Moreover, the *Devosia* strain A6-243 not only degraded DON, but also exhibited the ability to degrade 3-keto-DON (3-keto-deoxynivalenol) with the same product 3-epi-DON, indicating that DON epimerization by the *Devosia* strain A6-243 is a two-step enzymatic reaction. The most suitable conditions for the biodegradation process of the *Devosia* strain A6-243 were a temperature of 16–37 °C and pH 7.0–10, with 15–30 μM PQQ. In addition, the *Devosia* strain A6-243 was found to completely remove DON (6.7 μg/g) from DON-contaminated wheat. The results presented a reference for screening microorganisms with the ability of biotransform DON and laid a foundation for the development of enzymes for the detoxification of mycotoxins in grain and its products.

## 1. Introduction

Mycotoxins, including deoxynivalenol, trichothecenes, zearalenone, and aflatoxins, which are secondary metabolites that are mainly produced by *Aspergillus*, *Penicillium*, and *Fusarium* genera, are toxic to both humans and animals [1,2,3,4,5]. Among these, deoxynivalenol (DON, also known as vomitoxin) is the most abundant mycotoxin in cereal crops [6,7,8]. The accumulation of DON in the food chain threatens food safety and harms human health, causing appetite disturbance, immune suppression, nausea, and vomiting [3,9,10]. Therefore, the question of how to effectively diminish human exposure to DON needs to be urgently studied.

Several approaches have been applied to control and reduce DON contamination of crops, e.g., conventional physical and chemical detoxification as well as biodegradation by microorganisms [11]. Among these, biodegradation is considered to be the most environmentally friendly [2,8,12], as it produces the least amount of harmful substances and has less impact and a lower cost on food quality. The biodegradation of DON occurs mainly via oxidation and epimerization on C3 and de-epoxidation on C12–13, which are the principal moieties responsible for the toxicity of DON. The de-epoxidation of DON to de-epoxy-DON (DOM-1) by intestinal microorganisms requires anaerobic conditions that limit its application in agriculture and industry [4,7,13,14,15,16,17,18]. Oxidation and epimerization at C-3 transforms DON into 3keto and 3 epi DON, which are less toxic than DON [2,3]. To date, several microorganisms with the ability to biotransform DON have been successfully isolated, including *Aspergillus tubingensis*, *Nocardioides* sp., and *Devosia* sp. [17,19,20,21]. However, little is known about the enzymes involved in the transformation of DON and the underlying mechanisms. Recently, two enzymes called DepA and DepB were isolated from *Devosia* sp., which converted DON to 3-keto-DON and 3-keto-DON to 3-epi-DON, respectively [7]. This is the first time that research has proven that the complete isomerization process at the C3 position of DON is actually a two-part reaction involving two enzymes. Interestingly, the exogenous coenzyme pyrroloquinoline quinone (PQQ) is needed to assist DON biotransformation processes. For example, the enzymes DepA from *Devosia* mutants 17-2-E-8 and QADH from *Devosia* strain D6-9 both catalyze DON to form 3-keto-DON in the presence of PQQ. When no PQQ is present, they all lose their catalytic activity [22,23]. Since single bacterial strains cannot biotransform DON due to their weak ability to produce PQQ, the appropriate addition of exogenous PQQ may be beneficial for the screening of bacteria related to the biotransformation pathway. For some mixed bacteria cultures, one kind of bacteria produces cofactors to support another kind of bacteria with the enzymes, together building up the ability to biotransform DON.

In a previous study, we isolated mixed bacteria cultures of *Pseudomonas* sp. B6-24 and *Devosia* strain A6-243 [24], which were able to completely biotransform DON within 48 h. However, the *Pseudomonas* sp. B6-24 or *Devosia* strain A6-243 alone did not have the ability to biotransform DON. In the present study, different cofactors are added to isolate a single strain with the ability of biotransform DON from this mixed strain. On this basis, the biotransformation process is studied. Furthermore, the effect of the single strain on the biotransformation of DON in grains contaminated by *Fusarium graminearum* in practice is studied. Our research provides a method for screening and identifying new bacterial strains and enzymes that can biotransform DON.

## 2. Results

### 2.1. Isolation of DON-Transforming Bacteria from Soil Samples

**Figure 1:**
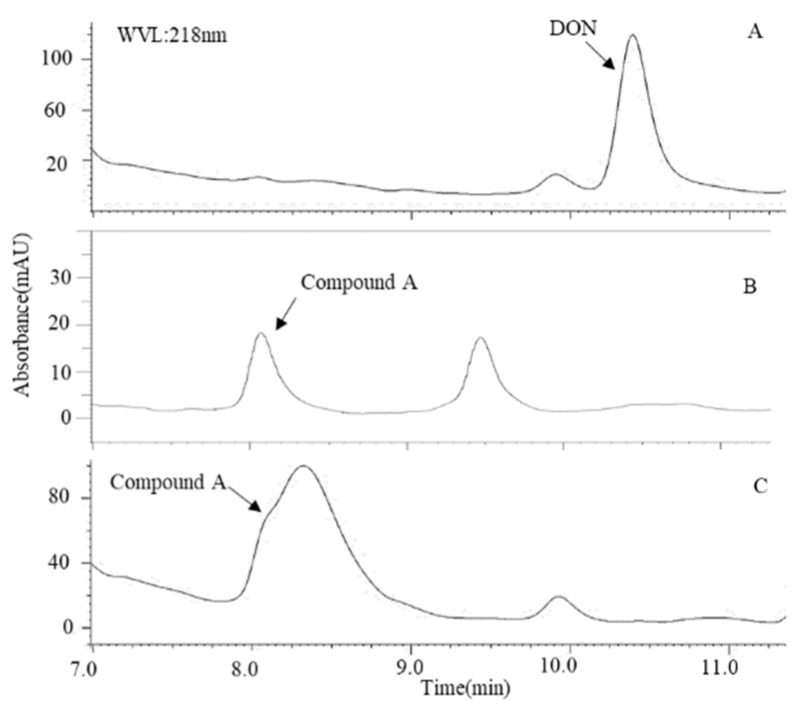
HPLC profile of DON biotransformation by the bacterial consortium of (**A**) the negative control, (**B**) *Pseudomonas* sp. B6-24 and *Devosia* strain A6-243, and (**C**) *Devosia* strain A6-243 with 100 μM of PQQ after 72 h of incubation. The initial DON concentration was 50 mg/L.

In a previous study, we isolated a bacterial consortium of *Pseudomonas* sp. B6-24 and *Devosia* A6-243 with the ability of biotransform DON [25]. The bacterial consortium of *Pseudomonas* sp. B6-24 and *Devosia* strain A6-243 was able to completely biotransform 50 μg/mL of DON within 72 h (Figure 1). Additionally, in the presence of 100 μM of PQQ, the *Devosia* strain A6-243 alone could biotransform 50 μg/mL of DON.

### 2.2. The Effect of CoFactors on DON Biotranformation by Devosia Strain A6-243 and Pseudomonas sp. B6-24

**Figure 2:**
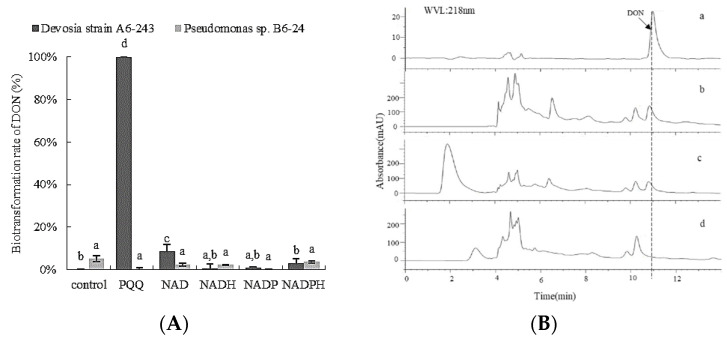
Biotransformation of DON by (**A**) *Devosia* strain A6-243 and (**B**) *Pseudomonas* sp. B6-24 using different cofactors. The bacteria were cultured in MMT (containing 100 μg/mL DON) for 72 h. (**B**) HPLC profile of DON standard: (a) concentration of DON after DON was added to be incubated with MMT; (b) concentration of DON after DON was cultured with DepA in MMT; (c) concentration of DON after DON was incubated with DepA and a culture supernatant of *Pseudomonas* sp. B6-24 in MMT (d).

To explore the effects of cofactors on the DON biotransformation process by the mixed bacteria culture, different cofactors (PQQ, NAD+, NADH, NADP+, and NADPH) were added to the culture during the cultivation. Apart from PQQ, other cofactors (NAD+, NADH, NADP+, and NADPH) showed no obvious effect on the biotransformation process by each single isolate of the mixed bacteria culture. The single isolate *Devosia* strain A6-243 supplied with exogenous PQQ was able to completely biotransform DON, while the strain showed no activity of biotransforming DON in the absence of PQQ (Figure 2A). By contrast, the single isolate *Pseudomonas* sp. B6-24 exhibited no activity of biotransforming DON in the presence of each cofactor (Figure 2A), indicating that *Pseudomonas* sp. B6-24 may act as a PQQ source in the bacterial consortium to trigger the biotransformation of DON by the *Devosia* strain A6-243 (Figure 2B).

### 2.3. Analysis of Transformation Conditions on DON Biotransformation

**Figure 3:**
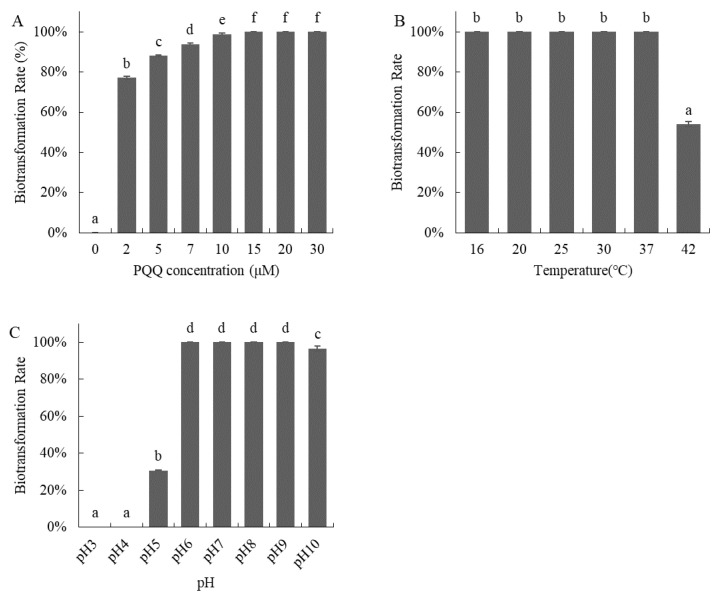
The effect of PQQ concentration (**A**), incubation temperature (**B**), and pH (**C**) on the biotransformation of DON by the *Devosia* strain A6-243. The difference of small letters a-f in the figure indicates significant difference between groups.

The effects of PQQ concentration, temperature, and pH on the biotransformation processes of the *Devosia* strain A6-243 were studied. The *Devosia* strain A6-243 biotransformed 100% of DON when 15–30 μM of PQQ was added (Figure 3A). As shown in Figure 3B, the *Devosia* strain A6-243 was able to completely biotransform DON in the range of 16–37 °C within 48 h, and the biotransformation slowed down at 42 °C. The *Devosia* strain A6-243 was able to biotransform DON completely in a from pH 6.0 to 9.0 within 48 h (Figure 3C), with 95% of DON being degraded at pH 10.0.

### 2.4. Characterization of DON Biotransformation Products by the Devosia Strain A6-243

The identification of DON biotransformation products is essential for determining the involved pathways. The scaled-up biotransformation of DON by the *Devosia* strain A6-243 led to the isolation and purification of the major biotransformation products.

**Figure 4:**
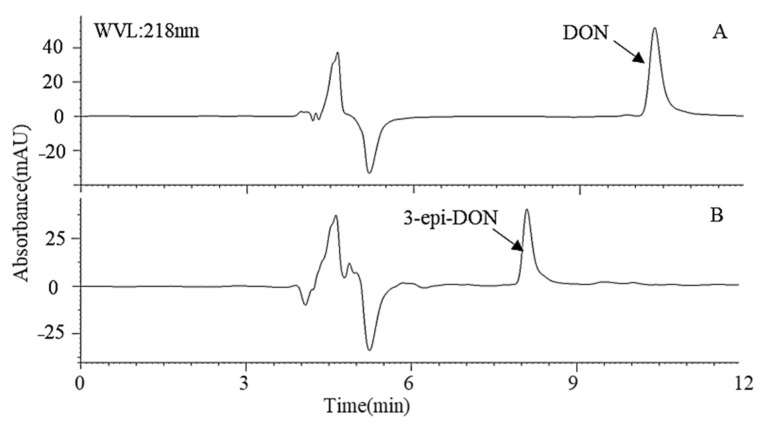
HPLC profile of the culture medium of the *Devosia* strain A6-243. (**A**) The cells of A6-243 were incubated with DON for 0 h. (**B**) The cells of A6-243 were incubated with DON for 72 h.

**Figure 5:**
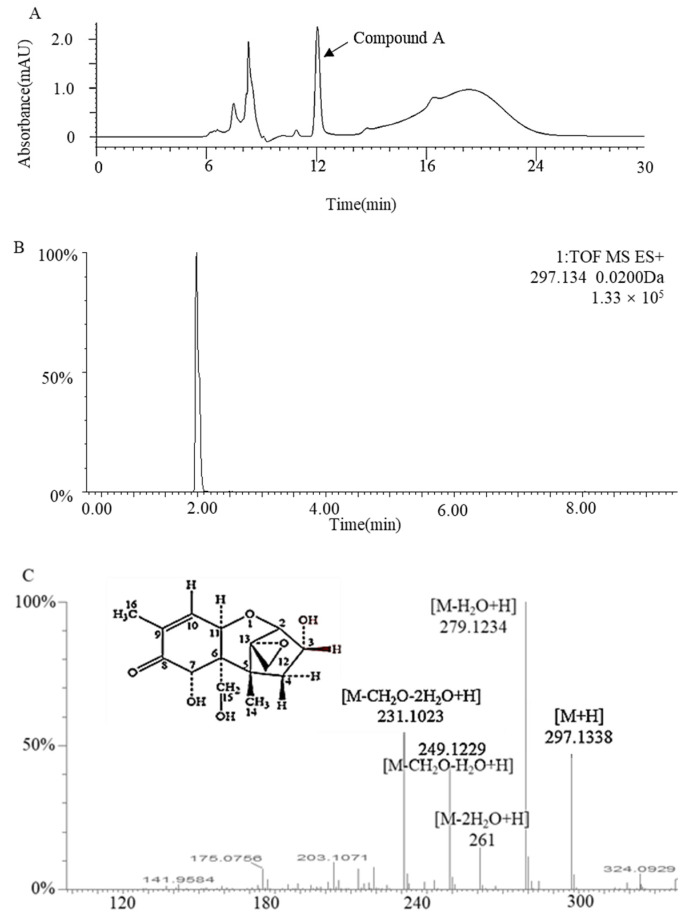
(**A**) Pre-LC profile of the *Devosia* strain A6-243 incubated with DON for 72 h. The 2D LC profile (**B**) and 2D MS spectra (**C**) of compound A. Purified compound A was analyzed with HQ-TOF-LC/MS/MS.

Compound A and DON were eluted at 8.2 min and 10.5 min, respectively, from a C18 column using a reverse-phase LC method in this study, indicating that compound A is more polar than DON (Figure 4B). According to a previous study [24], the retention time was the same as 3-epi-DON; then we used NMR and MS to confirm it. Through a pre-LC followed by analytical HPLC, one major intermediate, compound A, was revealed at 12.0 min of elution (Figure 5A). The purified compound A (5 mg) was characterized by LC/MS/MS and ^1^H and ^13^C NMR spectroscopy. According to its LC/MS/MS data (*m*/*z* 297.1338 ([M + H]^+^)), the molecular formula of the degradation product A was determined to be C_15_H_20_O_6_ (Figure 5). The characteristic ions of compound A at 297, 279, 261, 249, and 231 were the same as those of DON, but their relative abundances differed. Therefore, we speculated that the structure of compound A was 3-epi-DON. Further NMR data (Table 1) indicated the compound A was similar to that of DON, but the signal of H4 changed, meaning that the conformation of C3-OH had changed. These results confirmed that the DON biotransformation product was 3-epi-DON.

### 2.5. Characteristics of Deoxynivalenol Biotransformation by the Devosia Strain A6-243

**Figure 6:**
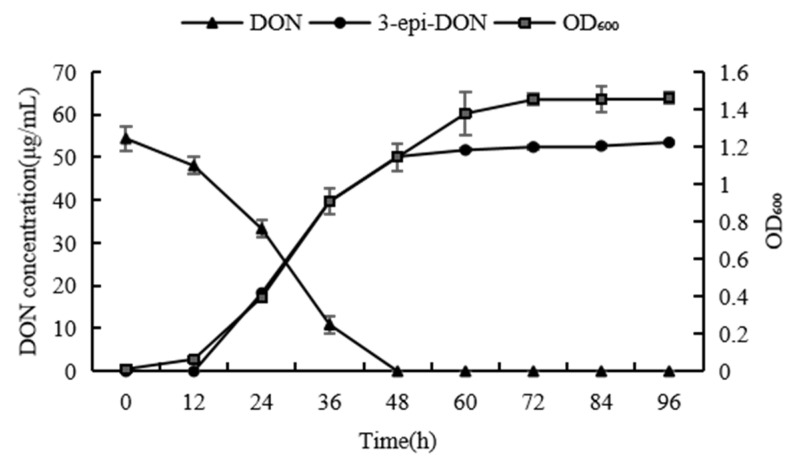
DON biotransformation and bacterial growth profiles of the *Devosia* strain A6-243. The curves represent changes in the DON/3-epi-DON concentration or the OD600 values of bacterial cultures.

To investigate the biotransformation of DON by the *Devosia* strain A6-243 over time, the decrease in the accumulation of DON and 3-epi-DON was studied. As shown in Figure 6, DON was biodegraded at 12 h of the reaction and completely converted into 3-epi-DON after 48 h of cultivation.

**Figure 7:**
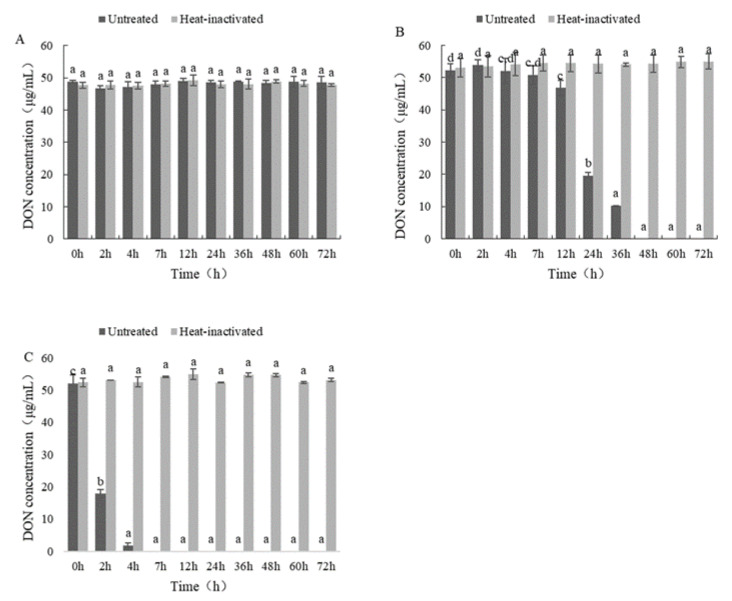
DON biotransformation by the culture supernatant (**A**), cell lysates (**B**), and cell debris (**C**) of the *Devosia* strain A6-243 at different time points at 37 °C. The difference of small letters a–d in the figure indicates significant difference between groups.

To define the components of the *Devosia* strain A6-243 that possess the ability to biotransform DON, the heat-inactivated and untreated supernatant, cell lysates, and cell debris of the *Devosia* strain A6-243 were investigated. The untreated cell lysates and cell debris were able to completely biotransform DON within 24 and 8 h, respectively (Figure 7). By contrast, the heat-inactivated supernatant, cell lysates, and cell debris did not show the ability of biotransform DON. The results indicated that DON is biotransformed and not absorbed by some bacterial structures.

### 2.6. Biotransformation of 3-Keto-DON by the Devosia Strain A6-243

**Figure 8:**
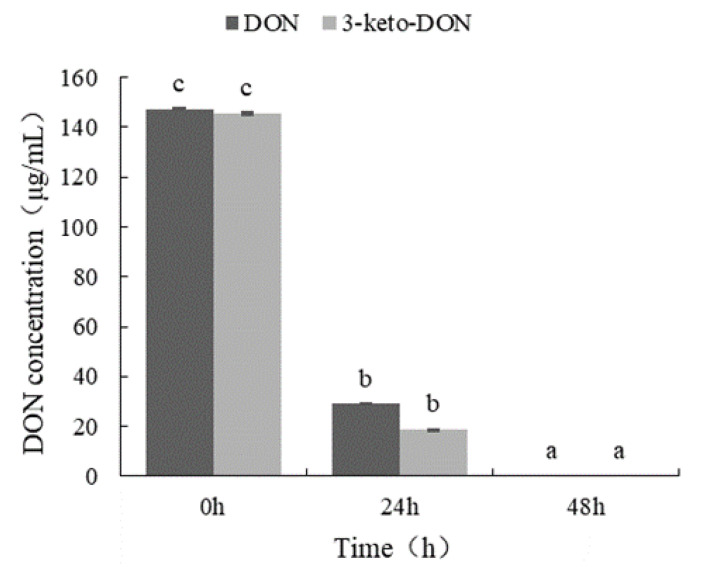
DON and 3-keto-DON biotransformation by the *Devosia* strain A6-243. The difference of small letters a-c in the figure indicates significant difference between groups.

The conversion of DON to 3-epi-DON via a two-step enzymatic reaction has been reported, where DON is first oxidized to 3-keto-DON and then reduced to 3-epi-DON [7]. Thus, to verify the epimerization process of DON by the *Devosia* strain A6-243, DON and 3-keto-DON were used as substrates in the process. As shown in Figure 8, DON and 3-keto-DON were completely biotransformed by the *Devosia* strain A6-243 within 48 h, suggesting that the DON epimerization of the *Devosia* strain A6-243 was also via a two-step enzymatic DON detoxification pathway.

### 2.7. Biotransformation of DON on Wheat Grain by the Devosia Strain A6-243

**Figure 9:**
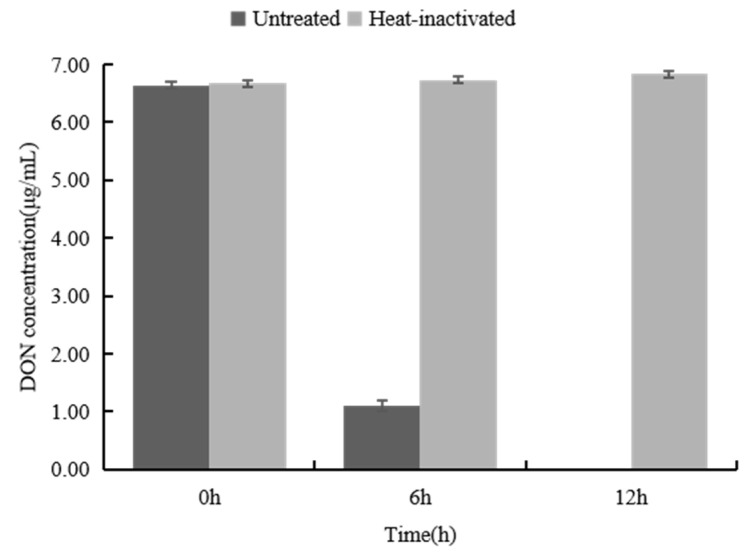
Biotransformation effect of the *Devosia* strain A6-243 on DON in contaminated wheat.

The DON biotransformation ability of the *Devosia* strain A6-243 in wheat grains was also studied. The DON content in wheat samples was 6.7 μg/g before treatment, and after 6 h incubation, the biotransformation rate of DON reached 85%. No DON could be detected after incubation with the *Devosia* A6-243 for 12 h, and the heat-inactivated sample showed no biotransformation ability after incubation for 12 h (Figure 9). These results establish that the strain D6-9 completely eliminates DON from wheat and can potentially be used for the detoxification of DON in agricultural products.

## 3. Discussion

This report proposes the use of PQQ to screen bacteria that can biotransform DON. This is extremely helpful for controlling screening costs and improving screening efficiency. The organisms known to produce PQQ are limited to certain Gram-negative bacteria, including *Acinetobactercalcoaceticus*, *Klebsiellapneu-moniae*, *Pseudomonas fluorescens*, *Methylobacteriumsquamosa*, *Methylobacteriumorgani-cophilum*, etc. [26]. Only *Pseudomonas* sp. B6-24 did not biotransform DON, even when the exogenous cofactors NAD (H), NADP (H), and PQQ were added. The ability of *Pseudomonas* sp. B6-24 to generate PQQ has also been confirmed by the NBT chemical method (data not shown) and HPLC (Figure 2B). The results showed that *Pseudomonas* sp. B6-24 had the ability to produce PQQ, which can be used for the biotransformation of DON with the *Devosia* strain A6-243. A previous study pointed out that the enzymes DepA and QADH derived from *Devosia* strains are both dependent on PQQ. Without PQQ, a simple enzyme does not have the ability to biotransform DON [22]. Interestingly, while we co-culture the purified DepA with *Pseudomonas* sp. in MMT media, DepA restores the biotransformation ability of DON, which indicates that PQQ produced by *Pseudomonas* sp. can be directly used by other types of enzymes. Considering the total amount of culture medium needed to screen strains, it is evidently impractical to add expensive exogenous PQQ to it. Therefore, the use of easy to cultivate and stable growth of PQQ-producing bacterial culture to replace pure PQQ as an exogenous additive is extremely meaningful for saving screening costs.

This research has also explored whether the mechanism of mixed bacteria to biotransform DON is the same as the epimerization with other bacteria [26,27]. Researchers have screened microorganisms that can isomerize DON from various environmental samples, such as soil *rhizobium* E3-39, *Devosia* mutants 17-2-E-8, and *Sphingomonas* S3-4 [7,28,29]. It has been reported that the epimerization of DON is a two-step enzymatic reaction by Devosia mutans 17-2-E-8, with 3-epi-DON as a final product [22,30]. Other studies have showed that isolated strains are able to either convert DON to 3-keto-DON or convert 3-keto-DON to 3-epi-DON [20,21,29]. In this study, a mixed bacteria culture of *Pseudomonas* sp. B6-24 and the *Devosia* strain A6-243 with DON-degrading activity was isolated, which completely biotransformed DON (100 μg/mL) within 48 h [25]. It biotransformed both DON and 3-keto-DON, with the final product being 3-epi-DON. This is different from previous mixed bacteria cultures, since most of these can only turn DON to 3-keto-DON, such as A culture D107 (mixture) [31]. In fact, the main single bacteria strains that can epimerize and degrade DON are *Devosia* sp., owing to the two types of enzymes classified as PQQ-dependent dehydrogenase and NADPH-dependent reductase, which have the functions of biotransforming DON to 3-keto-DON and biotransforming 3-keto-DON to 3-epi-DON, respectively [22,23,28]. Considering the relationship between the mixed bacteria, the biotransformation ability of each single isolate was studied. Since most bacteria can produce NADPH in their own metabolism, it was demonstrated that this strain lacks the ability to produce PQQ to activate the intrinsic enzymes with this function. In order to verify this hypothesis, exogenous PQQ was added to test whether it restored the degradation ability of the *Devosia* strain A6-243. As expected, when exogenous PQQ was added, this strain exhibited the ability to biotransform DON. These results indicate that the main biotransformation enzyme is present in the *Devosia* strain A6-243; subsequently, the whole-genome sequencing of *Devosia* strain A6-243 was performed and successfully discovered a PQQ-dependent alcohol dehydrogenase, which had an approximately 60% similarity to DepA (data not shown). This enzyme had the same effect as DepA and QADH and was able to biotransform DON into 3-keto-DON in the presence of exogenous PQQ. More similarly, there was at least one enzyme in the *Devosia* strain A6-243 that quickly biotransformed 3-keto-DON to 3-epi-DON, as we did not observe the accumulation of 3-keto-DON in the product. It seems that different kinds of *Devosia* have different biotransformation effects—for example, *Devosia* insulae A16 can only biotransform DON to 3-keto-DON [21]. These differences may be caused by the type and quantity of dehydrogenase and aldehyde ketone reductase in bacteria. From these results, it can be found that the lack of a PQQ synthesis gene limits the detoxification effect of some *Devosia* strains, leaving some strains with an undiscovered detoxification potential.

In addition, for some mixed cultures, the inherent biotransformation mechanism remains to be studied. Maybe some of their enzymes are not PQQ dependent, or some bacteria may have one-step detoxification enzymes. Sato [17] reported nine *Nocardioides* sp. and five *Devosia* sp. that can biotransform DON to 3-epi-DON. Although the mechanism by which mixed bacteria biotransform toxins was not clarified, the internal mechanism should be the same. Previously, Zhai [24] screened a mixed culture of two novel strains named *Pseudomonas* sp. Y1 and *Lysobacter* sp. S1, which showed the sustained transformation of DON into the metabolite 3-epi-deoxynivalenol. Generally, *Pseudomonas* sp. has potential synthetic function with PQQ, but there are fewer reports regarding the ability of *Lysobacter* sp. to biotransform DON. It seems that the mechanisms by which these two mixed bacteria biotransform vomiting are different.

In this paper, the DON-transforming activity of the *Devosia* strain A6-243 was found in cell debris and cell lysates, but heat-inactivated supernatant, cell debris, and cell lysates showed no DON-transforming activity. Thus, an enzymatic reaction was involved in this biotransformation process, and the epimerase was mainly located in the cell membrane. Furthermore, the biotransformation conditions were also investigated, and the results showed that the most suitable conditions for the biotransformation of the *Devosia* strain A6-243 were at 37 °C and pH 7.0, which was similar to that of the *D.* insulae A16 reported by Wang et al. [21]. Finally, the DON biotransformation ability of the *Devosia* strain A6-243 in DON-contaminated wheat grains was investigated. The results showed that the *Devosia* strain A6-243 exhibited a strong DON-transforming ability and could completely biotransform DON (6.7 μg/g) in DON-contaminated wheat grains. These results demonstrated that the *Devosia* strain A6-243 could completely biotransform DON in wheat grains, and it exhibited an enormous potential to be used as an additive in crops to address DON contamination problems under empirical field conditions.

## 4. Conclusions

Previously, mixed bacterial cultures of *Pseudomonas* sp. B6-24 and *Devosia* strain A6-243 with DON biotransformation activity were isolated. In order to further analyze which bacteria played the main role in the DON biotransformation by *Pseudomonas* sp. B6-24 and *Devosia* strain A6-243, the DON-transforming ability of each single isolate was studied. The results showed that each single isolate lost its DON-transforming ability, although there was no observable detrimental effect on bacterial cell growth. Interestingly, the *Devosia* strain A6-243 recovered its DON biotransformation activity with the assistance of the exogenous cofactor PQQ. The *Devosia* strain A6-243 was not only able to biotransform DON, but also exhibited 3-keto-DON biotransformation activity, with epi-DON being the main product, indicating that a two-step enzymatic reaction was involved in this biotransformation process. These products have been shown to be less toxic [2]. The most suitable conditions for the biodegradation process of the *Devosia* strain A6-243 were at 16–37 °C and pH 7.0–10 with 15–30 μM PQQ. Moreover, the *Devosia* strain A6-243 showed a strong DON detoxification ability in DON-contaminated wheat and DON was converted to 3-epi-DON in wheat grains (6.7 μg/g). These results indicate the potential of the use of the *Devosia* strain A6-243 as a DON biotransformation agent to control DON contamination in cereal grains.

## 5. Materials and Methods

### 5.1. Chemicals and Media

DON and 3-keto-DON standards were purchased from TripleBond (Guelph, ON, Canada). Mineral salt medium (MM) was formulated as described previously [24], with slight modifications (1.6 g/L Na_2_SO_4_·12H_2_O, 1.0 g/L KH_2_PO_4_, 0.5 g/L MgSO_4_·7H_2_O, 0.5 g/L NaNO_3_, 0.5 g/L (NH_4_)_2_SO_4_, and 0.025 g/L CaCl_2_). Mineral salt with tryptone medium (MMT) was composed of basic MM supplemented with 2 g of tryptone.

### 5.2. Apparatus and Conditions for High-Performance Liquid Chromatography and Preparative Liquid Chromatography

A high-performance liquid chromatography (HPLC) analysis was conducted using an Ultimate 3000 station (Thermo, Shanghai, China) equipped with an ultraviolet detector. The chromatographic column used a 5HC-C18 analytical column (250 mm × 4.6 mm, Agilent). Isocratic elution was performed with methanol and water (30:70, *v*/*v*) at a flow velocity of 0.6 mL/min. The injection volume was 20 µL. Under this condition, DON was eluted at 10.5 min, and 3-epiDON at 8.0 min. Preparative liquid chromatography (Pre-LC) was performed using a Waters 600 controller (Waters, Massachusetts, USA) equipped with an ultraviolet detector. The chromatographic column was performed using an XBridge C-18 column (19 mm × 150 mm, film thickness 5 μm; Waters). Isocratic elution was performed with methanol and water (30:70, *v*/*v*) at a flow velocity of 4 mL/min. The injection volume was five milliliters. Under these conditions, DON was eluted at 20.0 min, and 3-epi-DON at 12.0 min. HPLC and pre-LC were both performed at a wavelength of 218 nm.

### 5.3. DON-Transforming Activity Analysis of Deoxynivalenol-Degrading Bacteria

Mixed bacteria cultures of *Pseudomonas* sp. B6-24 and *Devosia* A6-243 with a high DON-transforming activity were used for enrichment. An aliquot (20 μL) of the culture was transferred to 2 mL of MMT with 50 μg/mL of DON and cultured at 37 °C and 180 rpm for 72 h. After 72 h of incubation, an aliquot (20 μL) of the culture was transferred to fresh MMT. In negative controls, no mixed bacteria were added. Each procedure was repeated ten times, and the DON-transforming activity of the culture was examined by high-performance liquid chromatography (HPLC) before every sample was transferred to fresh MMT.

### 5.4. The Effect of CoFactors on DON Biotransformation by the Devosia Strain A6-243 and Pseudomonas sp. B6-24

In order to study the effect of cofactors on the biotransformation of DON by *Devosia* strain A6-243 and *Pseudomonas* sp. B6-24, the *Devosia* strain A6-243 and *Pseudomonas* sp. B6-24 were inoculated into MMT medium at 37 °C for 72 h. The seed culture (20 μL) of the *Devosia* strain A6-243 and *Pseudomonas* sp. B6-24 was inoculated into 2 mL of MMT medium (100 μg/mL DON) containing different cofactors (PQQ, NAD+, NADH, NADP+, and NADPH, 100 μM). Then, each sample was cultured at 37 °C and 180 rpm for 72 h, and the DON concentrations were analyzed by HPLC.

In order to study whether PQQ was produced in the culture of *Pseudomonas* sp. B6-24, the NBT chemical method was used to detect PQQ in the culture medium. *Pseudomonas* sp. B6-24 were inoculated in an MMT medium at 37 °C for 72 h, and the supernatant was collected by centrifuge at 8000× *g* rpm for 10 min. The PQQ was detected by NBT chemical method. Briefly, an aliquot of 10 µL of supernatant was added to buffer containing 40 µL 20 mM PBS (pH = 7.0), 90 µL 20 mM Gly-KOH (pH = 10.0), and 10 µL 3.6 mM NBT. A buffer containing 10 μL PQQ was set as the positive control. Then, the reaction was incubated at 30 °C for 1 h in the dark. The absorbance was determined at 535 nm. DepA [22], a PQQ-dependent enzyme, was expressed and purified in our laboratory, and was used to further verify the ability of *Pseudomonas* sp. B6-24 to produce PQQ. The *Pseudomonas* sp. B6-24 supplemented with 500 μM DON was incubated with the DepA (30 μg) and Ca^2+^ (10 μM) at 37 °C for 48 h. The mixture without *Pseudomonas* sp. B6-24 was used as a negative control.

### 5.5. Effect of Transformation Conditions on DON Biotransformation

Various cultural conditions, such as PQQ concentration (0 μM, 10 μM, 15 μM, 20 μM, and 30 μM), incubation temperature (16 °C, 20 °C, 25 °C, 30 °C, 37 °C, and 42 °C), and medium pH (3.0, 4.0, 5.0, 6.0, 7.0, 8.0, 9.0, and 10.0), were optimized in order to enhance the DON-biotransforming ability of the *Devosia* strain A6-243. The one-factor-at-a-time method was used to optimize the process parameters. The reactions were incubated at 180 rpm for 48 h and the DON concentrations were analyzed by HPLC.

### 5.6. Characterization of DON Biotransformation Products

To investigate the major products of DON biotransformation by the *Devosia* strain A6-243, the strain was inoculated into the medium and cultured at 37 °C and 180 rpm for 72 h. The culture was harvested by centrifugation at 4 °C and 8000× *g* rpm for 10 min, and the precipitate was washed twice in a buffer containing 50 mM of K_2_HPO_4_-KH_2_PO_4_, pH = 7.0, 300 mM NaCl, and subjected to ultrasonication for cell disruption. The disrupted cells were removed by centrifugation at 4 °C for 10 min, and the supernatant was collected and incubated with 10 mg of DON and 20 μM PQQ at 37 °C for 72 h. The supernatant was injected into the pre-LC column, and the DON-transforming products were identified according to retention times and verified by HPLC. The collected fractions were concentrated using a rotary evaporator and dissolved in water. Then, the products were extracted three times, each time using ethyl acetate. The upper layer was collected and dried using a nitrogen stream to remove the ethyl acetate. Finally, 5.0 mg of pure compound A was obtained. The structure of compound A was determined by G2-XS QTof- LC–MS system (Waters, ON, USA). and an NMR spectrometer (Bruker 400 M, ON, Germany).

### 5.7. Analysis of 3-Keto-DON Biotransformation by the Devosia Strain A6-243

In order to analyze the DON metabolic pathway of the *Devosia* strain A6-243, the strain was incubated with DON or 3-keto-DON. The *Devosia* strain A6-243 was inoculated into 2 mL of an MMT liquid medium and incubated for 48 h, and then DON and 3-keto-DON (150 μg/mL) were added to the culture, respectively. The culture was incubated at 37 °C for different lengths of time, and acidified methanol was added to terminate the reaction. The concentration changes of DON and 3-keto-DON in the reaction system were detected by HPLC.

### 5.8. Biotransformation of DON on Wheat Grain by the Devosia Strain A6-243

Wheat cultivar Zhengmai9023 grains (autoclaved for 18 min at 121 °C) that had acquired *Fusarium* head blight (FHB) pathogens were used to measure the DON-transforming ability of the *Devosia* strain A6-243, with heat-inactivated *Devosia* strain A6-243 cells used as a negative control. After 72 h of culture in MMT, the cells were harvested by centrifugation and added to reactions containing 2× *g* of autoclaved wheat and 10 μM of PQQ in 2 mL of a sodium phosphate buffer (50 mM, pH 7.0). The reactions were incubated at 37 °C for 12 h. The samples were dried and ground; then, the DON was extracted with acetonitrile and water and assayed by HPLC [23].

### 5.9. Statistical Analysis

The experimental results were expressed as the means and standard deviations (SDs) of the replicate determinations. All statistical analyses were carried out using Origin 2021.

## Figures and Tables

**Table 1:** ^1^H and ^13^C NMR data of product A and DON standards.

Position	^1^H-NMR δH, ppm (mult, J in Hz)		^13^C-NMR δC (ppm)	
	DON	Product A	DON	Product A
2	3.65 (d, 4.5)	3.72 (s)	80.9	80.9
3	4.56 (dt, 4.3, 4.4, 9.1)	4.58 (d, 6.0)	69.3	69.3
4	2.22 (dd, 4.3, 14.8)	2.33 (dd, 7.6, 7.6)	43.3	43.3
	2.10 (d, 4.0)	2.22 (s)		
5			46.6	47.6
6			52.1	52.1
7	4.85 (d, 1.8)	4.92 (s)	74.6	74.6
8			200.3	200.0
9			136.1	136.1
10	6.63 (dd, 1.5, 5.9)	6.57 (dd, 1.6, 5.9)	138.6	138.6
11			70.5	69.3
12			65.7	65.7
13	3.10 (d, 4.3)	3.19 (d, 4.2)	47.6	47.6
	3.17 (d, 4.2)	3.14 (d, 4.2)		
14	1.15 (s)	1.21 (m)	14.5	14.5
15			62.7	62.7
16	1.94 (s)	1.91 (s)	15.5	15.5

## Data Availability

Not applicable.
